# Using Dihydrazides as Thermal Latent Curing Agents in Epoxy-Based Sealing Materials for Liquid Crystal Displays

**DOI:** 10.3390/polym13010109

**Published:** 2020-12-29

**Authors:** Jun Hyup Lee

**Affiliations:** Department of Chemical Engineering, Soongsil University, Seoul 06978, Korea; junhyuplee@ssu.ac.kr; Tel.: +82-2-829-8329

**Keywords:** dihydrazide, epoxy resin, latent curing agent, liquid crystal display, sealing material

## Abstract

In this study, highly adhesive epoxy-based sealing materials for liquid crystal (LC) displays were fabricated using different types of dihydrazides as thermal latent curing agents. Their curing characteristics, mechanical properties, LC contamination levels, and electro-optical characteristics were investigated depending on the chemical structure of dihydrazides. The epoxy-based sealing material containing a dihydrazide derivative with a bulky heterocyclic ring afforded a high heat curing conversion of 90.4%, high adhesion strength of 54.3 kgf cm^−2^, and a high elongation of 57.3% due to the relatively low melting characteristic under heat treatment compared to those involving dihydrazides with short aliphatic or aromatic spacers. In addition, the proposed sealing material exhibited an extremely low LC contamination level of 9 µm, which is essential to the successful operation of LC displays. With respect to electro-optical properties of the LC device, it was found that a dihydrazide derivative with a bulky heterocyclic ring afforded a normal voltage-dependent transmittance curve and fast response time due to the prevention of abnormal homogeneous LC alignment. This study developed highly adhesive and robust epoxy-based sealing materials based on the use of dihydrazides as thermal latent curing agents for advanced LC displays.

## 1. Introduction

Of late, liquid crystal displays (LCDs) are finding wide use in various applications, including in tablets, monitors, and televisions. Owing to recent developments in television manufacturing technologies, the bezel, which is the plastic part around the screen, is getting thinner because of users’ preference for larger screens. For this reason, LCD sealing materials with improved properties such as higher adhesive strength, lower moisture permeability, and greater contact contamination resistance need to be developed. For increasing the adhesive strength, several methods, such as polymer blending, curing with polythiourethanes, and using epoxy-acrylate oligomers, have been proposed [1,2,3]. The blending of the epoxy with lignin can increase the curing degree and improve the shear strength of the epoxy resin [1]. Further, polyurethanes have been used as hardeners for increasing the adhesive properties and lowering the curing temperature [2]. Researchers also synthesized partially acrylated resins based on epoxy acrylate oligomers for use as dual-curing resins and investigated their mechanical properties [3]. However, these studies utilized specific polymers or modified epoxy resins for high adhesion properties.

Epoxy resins show several desirable properties, such as high chemical and solvent resistances and excellent adhesive properties [4,5,6,7]. Hence, epoxy resins are widely used in protective coatings and electrical laminates as well as adhesives. In particular, adhesives based on epoxy resins are highly suited for bonding with various surface types, such as steel, plastic, wood, and composites, owing to the formation of a network-like structure after the curing of the epoxy resin [8,9]. In these cases, the epoxy resin is mostly used with other polymers or plasticizers such as rubber, polyurethane, or phenolic acid [10,11,12,13,14]. Further, in order to aid the formation of the network-like structure, a curing agent is usually employed with the epoxy resin. The curing agent contains amine groups that can react with the epoxy resin under certain curing conditions, leading to the formation of a network-like structure through a reaction involving the opening of the epoxy ring. From a mechanical properties viewpoint, it has been shown that a mixture of metaphenylene diamine (MPDA) and N-(3-phenoxy-2-hydroxypropyl)-1,3-benzenediamine (NPHB) is highly suited for use as a curing agent [15]. The epoxy resin and hardener were cured under different curing conditions (heating temperature and duration) in order to determine the optimal conditions for ensuring good mechanical properties. Knowing the curing behavior of epoxy resins is essential for determining the optimal curing and processing conditions, with the behavior of the resins depending on the curing agent used [16,17].

In order to improve the tensile strength and humidity resistance of the sealing materials used for LCD panels, a few researchers have used ultraviolet (UV)-curable adhesive sealants. These were synthesized using a UV-curable resin based on a commercial epoxy resin through the alkylation method [18,19]. Using this method, a photoinitiator is mixed with a synthesized UV-curable resin, and the resulting sealing material is solidified under UV irradiation (365 nm) using a commercial UV lamp, producing a formed sealant that exhibits high tensile strength. However, there have been few studies on the heat curing of LCD sealing materials using a latent curing agent as the hardener [20,21]. Latent curing agents show high activity under external stimulation. Thus, using such latent curing agents as the hardener in the sealant system should lead to the realization of a one-pot curing mixture for display applications.

In this study, we fabricate highly adhesive epoxy-based sealing materials for LCDs using different types of dihydrazides as the thermal latent curing agent. The dihydrazide compounds contain the reactive amine groups of C=ONHNH_2_ and have either aliphatic or aromatic spacers; thus, they can react with the epoxide ring of epoxy resins. Dihydrazides with different backbone structures have different curing effects on epoxy resins, thereby leading to the difference in adhesion properties. Further, because dihydrazide derivatives show latent curing characteristics under high temperature operation [20], they are highly suited for the fabrication of LCD devices. Thus, we use four types of dihydrazides derivatives including adipic acid dihydrazide (ADH), isophthalic dihydrazide (IDH), sebacic dihydrazide (SDH), and 4-isopropyl-2,5-dioxoimidazolidine-1,3-di(propionohydrazide) (VDH) as thermal latent curing agents for epoxy-based sealing materials, and we analyze the physical properties of all the cured resin samples in order to evaluate their suitability for use as adhesive sealing materials for LCD panels. We also examine the curing conditions, curing degree, adhesive properties, and LC contamination levels of the fabricated epoxy/dihydrazide adhesives. Finally, we investigate device properties such as the voltage-transmittance characteristics and the response time.

## 2. Materials and Methods

### 2.1. Materials

**Figure 1:**
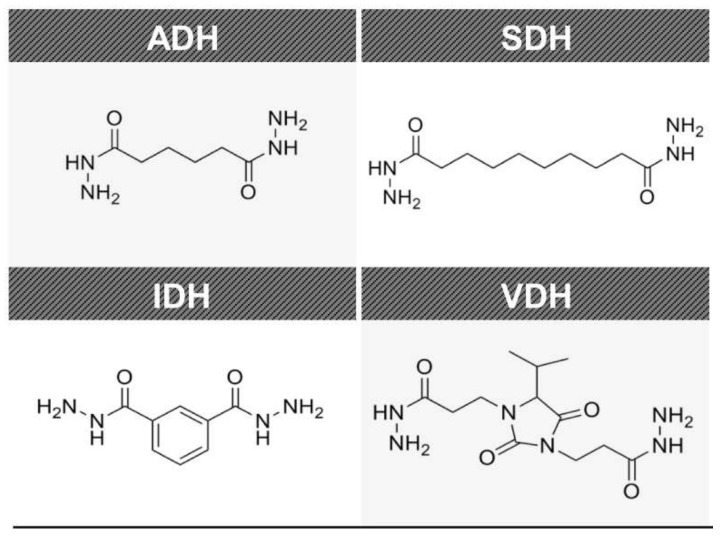
Chemical structures of ADH, IDH, SDH, and VDH.

As stated above, four types of dihydrazide derivatives, including adipic acid dihydrazide (ADH, Tokyo Chemical Industry, Tokyo, Japan), isophthalic dihydrazide (IDH, Tokyo Chemical Industry, Tokyo, Japan), sebacic dihydrazide (SDH, Tokyo Chemical Industry, Tokyo, Japan), and 4-isopropyl-2,5-dioxoimidazolidine-1,3-di(propionohydrazide) (VDH, Ajinomoto Fine-Techno, Kanagawa, Japan), were utilized in this study and demonstrated in Figure 1. The epoxy resin was a bisphenol A-type epoxy resin with an epoxy equivalent weight of 184-190 g/eq (YD-128, Kukdo Chemical, Seoul, Korea). The acrylate resin of bisphenol A glycerolate dimethacrylate (BisGMA) was received from Sigma-Aldrich (St. Louis, MO, USA). The photoinitiator, Irgacure 651, was purchased from Ciba Specialty Chemicals (Basel, Switzerland). Fumed silica with an average diameter of 250 nm was obtained from Sigma-Aldrich (St. Louis, MO, USA).

### 2.2. Preparation of Epoxy-Based Sealing Materials

The epoxy resin system for LC displays was prepared by physical blending using a rotation and revolution mixer (AR-100, Thinky, Tokyo, Japan). The YD-128 epoxy resin (40.0 g) and BisGMA acryl resin (40.0 g) were blended with the fumed silica (16.0 g) and a photoinitiator (2.5 g) at 2000/1300 rpm for 15 min. Then, the dihydrazide (1.5 g) was added to this mixture and blended at 2000/1300 rpm for 5 min. After the completion of the mixing process, the epoxy mixture was defoamed in a vacuum oven at 25 °C for 2 h. This yielded a white viscous liquid of the desired adhesive.

### 2.3. Characterization

To analyze the phase transition behaviors of the used dihydrazide compounds, we used differential scanning calorimetry (DSC; TA instrument, Q20, New Castle, DE, USA). Each dihydrazide compound of 10 mg was heated from 50 to 300 °C at the rate of 10 °C min^−1^ and subjected to DSC scanning. Thermogravimetric analysis (TGA) was performed using a thermal analyzer (TA instrument, SDT-Q600, New Castle, DE, USA) in the temperature range from 20 to 800 °C. Further, we used Fourier-transform infrared spectroscopy (FTIR; Jasco, FT/IR-460 Plus, Easton, PA, USA) to determine the curing conversion ratio of each epoxy resin system under the curing conditions corresponding to LCD fabrication. Each epoxy sealing material was cured on a glass substrate under UV radiation with a radiant fluence of 3.0 J cm^−2^ and then heat-cured at 120 °C for 1 h.

In order to determine the adhesive strength of the epoxy-based sealing materials, the resins were cured between two indium tin oxide (ITO) glass substrates. The two ITO glass substrates were assembled in a cross-wise manner, and the diameter of the adhesive sample used was 0.2 cm. This assembly was exposed to UV radiation with an intensity of 20 mW cm^−2^ for 150 s. After the UV curing process, the assembly was heat-cured in an oven at 120 °C for 1 h. The resulting sealant sample had a circular shape with a thickness of 5 µm. A similar assembly was used to analyze the lap shear strength. In this case, the glass substrates were assembled in a straight manner, while all the other conditions were the same. The adhesive and lap shear strengths were measured with a universal testing machine (UTM; LLOYD, LR-5K, Bognor Regis, England) at room temperature according to the ASTM C663 and D3165 standards; the crosshead speed used was 1.3 mm min^−1^ [22,23,24].

To evaluate whether the epoxy sealing materials contaminated LCs on contact, we fabricated test LC cells using ITO glass substrates covered with a layer of a polyimide (PI; JSR, AL607XX, Tokyo, Japan) using a spin coater. The PI layer on the ITO glass substrate was subjected to primary baking on a hotplate at 80 °C for 10 min. It was then hard-baked in an oven at 230 °C for 1 h. Next, the prepared sealing adhesive was cured between two PI-layer-coated ITO glass substrates arranged in a cross-wise manner, and LCs (*T*_NI_ = 75 °C, Δ*n* = 0.095, Δ*ε* = −3.1) were injected between the substrates. The curing conditions for the adhesive were the same as those during the adhesive strength measurements. To confirm whether the LCs had been contaminated, the fabricated sample cell was examined using polarized optical microscopy (POM; Olympus, BX51, Tokyo, Japan).

The electro-optical characteristics (including the voltage–transmittance curves and response times) of the LC devices containing epoxy resin systems were measured with an electro-optical measurement system. The system consisted of an optical table (Future Science, FT-R120, Seoul, Korea) equipped with a 632 nm HeNe laser (JDSU, 1135P, Milpitas, CA, USA), a photodetector (EOT, ET-2000, Edinburgh, United Kingdom), a function generator (Agilent, 33210A, Santa Clara, CA, USA), and an oscilloscope (Tektronix, TBS1062, Beaverton, OR, USA).

## 3. Results and Discussion

### 3.1. Curing Anylysis of Epoxy-Based Sealing Materials

**Figure 2:**
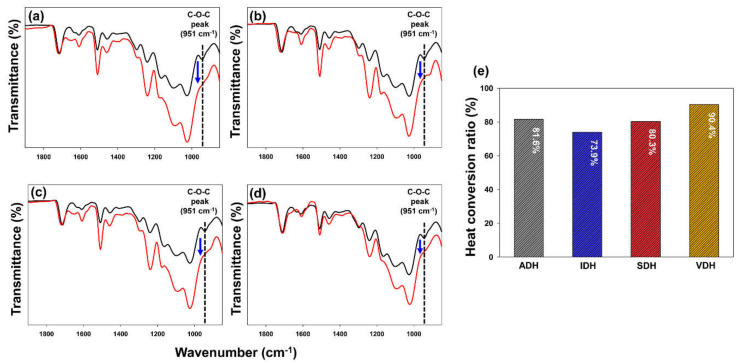
FTIR spectra of various epoxy resin systems containing (**a**) ADH, (**b**) IDH, (**c**) SDH, and (**d**) VDH before (black line) and after (red line) heat treatment. (**e**) Conversion ratios of epoxy-based sealing materials after heat-curing process.

The heat curing behaviors of the epoxy resin systems, including different types of dihydrazides, were analyzed using FTIR in the attenuated total reflection mode. We compared the conversion ratios of the epoxy resin systems under the industrial curing conditions for LCDs based on the changes in the area of the C-O-C epoxide peak. In the prepared epoxy sealing materials, only the NH_2_ group of the dihydrazides would interact with the oxirane rings of the epoxy resin. Thus, we compared the area of the epoxide-ring-related peak between 921 and 968 cm^−1^ [21], as shown in Figure 2. The heat curing process for each epoxy resin system was performed at 120 °C for 1 h. We calculated the conversion ratios of the epoxy sealing materials in terms of percentage values. The epoxy resin system formed using VDH showed the highest heat conversion ratio, which was 90.4%. In contrast, the epoxy resin system formed using IDH as the hardener showed the lowest conversion ratio, which was 73.9%. Finally, the epoxy resin systems formed using SDH and ADH exhibited similar conversion ratios, which were 80.3% and 81.6%, respectively.

**Figure 3:**
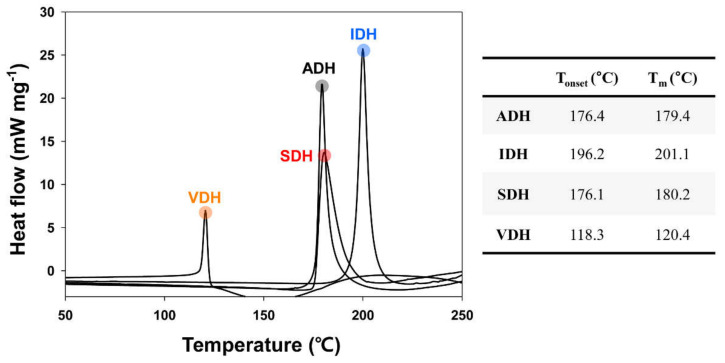
Differential scanning calorimetry (DSC) thermograms of various dihydrazide compounds at heating rate of 10 °C min^−1^.

To investigate the difference in heat conversion ratio under the same curing condition, DSC measurement was performed on the dihydrazide compounds. The endothermic heat curves of the various dihydrazide derivatives obtained at a heating rate of 10 °C min^−1^ are shown in Figure 3. The solid dihydrazide compound as a thermal latent curing agent can efficiently react with the epoxy resin at elevated temperatures after melting transition [20]. The melting points (T_m_) were obtained based on the peak temperature of the endothermic transition. The results indicated that the used dihydrazide derivatives exhibit the potential of latent heat curing agent due to the high-temperature melting transitions above 100 °C. As mentioned above, these dihydrazide compounds showed nonreactivity under a normal room temperature condition but exhibited high reactivity under high curing temperatures. While the IDH compound showed the highest melting transition at 201.1 °C due to the rigid aromatic spacer, the VDH compound exhibited the lowest melting point at 120.4 °C due to presence of the bulky heterocyclic ring. Notably, the melting transition of VDH is very close to the thermal curing temperature (120 °C) for LCD fabrication. In addition, it was confirmed that the VDH compound with a relatively low melting point provided a high heat conversion ratio of epoxy resin due to the efficient melting transition during the high-temperature curing process. Therefore, under industrial curing conditions, the VDH compound as a thermal latent curing agent will show effective curing performance for epoxy-based sealing materials.

**Figure 4:**
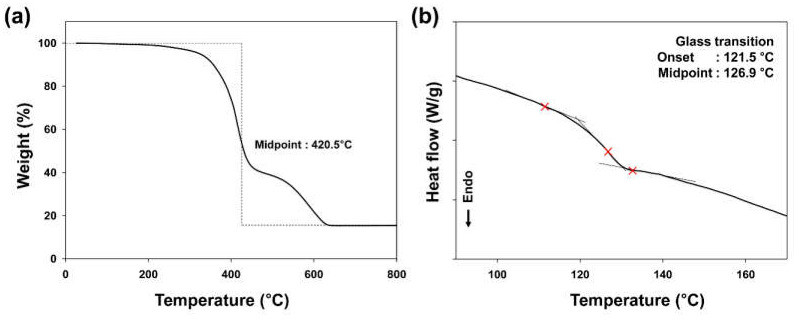
(**a**) TGA and (**b**) DSC thermograms of epoxy resin system with VDH.

Figure 4 showed the TGA and DSC thermograms of the epoxy resin system with VDH. Since the thermal curing temperature for LCD fabrication is 120 °C, the epoxy-based sealing materials should have high thermal stability over 120 °C [25,26]. As shown in Figure 4a, no noticeable weight loss was observed even at 200 °C, indicating the high thermal stability of the prepared sealant for a successful fabrication and operation of an LCD [27,28]. In addition, as shown in Figure 4b, the glass transition of the epoxy resin system was observed at approximately 125 °C, and it was close to the heat curing temperature of 120 °C, suggesting that a high chain mobility of epoxy resin can promote the curing reaction with dihydrazide compounds.

### 3.2. Mechanical Properties of Epoxy-Based Sealing Materials

**Figure 5:**
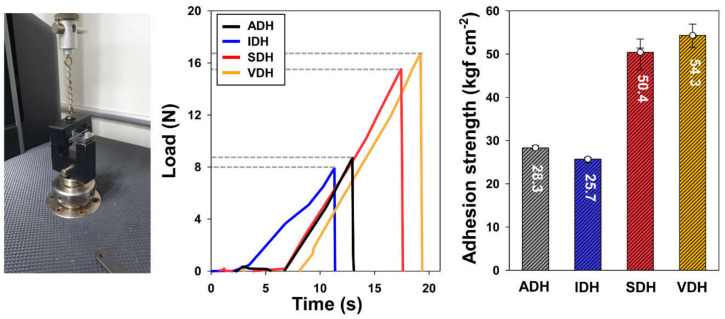
Results of pull-off test for various epoxy resin systems.

The results of the pull-off test for each epoxy resin system are shown in Figure 5. The adhesive strength of the epoxy-based sealing material with VDH included as the hardener was the highest at 54.3 kgf cm^−2^. Because VDH melts at 120 °C, its diffusion rate is higher, and this resulted in an epoxy resin system with the greatest reactivity among the others tested. As a result, there was an increase in the number of cross-linking sites between the epoxy and the dihydrazide when it was cured under the conditions used in this study. On the other hand, since IDH has the highest melting point at 201 °C, the lowest adhesion strength (25.7 kgf cm^−2^) was obtained. Moreover, the bulkier the spacer structure of the dihydrazide used, the less brittle the cured epoxy system will be when it is detached. This was evident from a comparison of the results for the ADH and SDH epoxy resin systems. The adhesive strength of the epoxy resin system containing ADH was a low value of 28.3 kgf cm^−2^. In contrast, the epoxy resin system containing SDH showed a high adhesion strength of 50.4 kgf cm^−2^. Because the alkyl chain of SDH is much longer than that of ADH, the adhesive force of the cured epoxy resin system based on SDH was higher.

**Figure 6:**
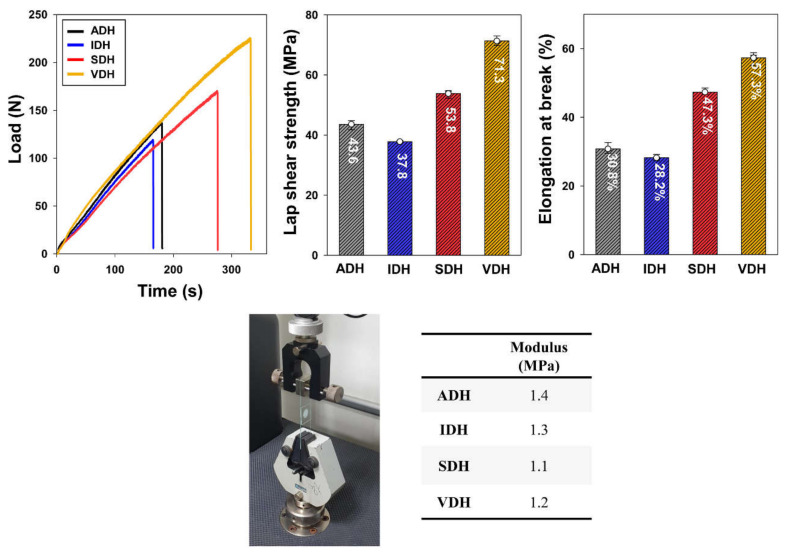
Results of lap shear test for various epoxy resin systems and their modulus values.

The results of the lap shear strength measurements are shown in Figure 6. The lap shear strength and elongation at the break of the epoxy-based sealing materials are in accordance with the adhesive strength in the pull-off test. The SDH system exhibited a higher shear strength than the ADH system, which indicates that with an increase in the alkyl chain length, the internal stress is reduced because of chain flexibility; this in turn improves the crack propagation resistance. For this reason, the brittleness and impact sensitivity of the cured epoxy resin system with ADH were higher, and its shear strength was lower than that of the system with SDH. Further, in addition to the alkyl chain length, the bulkiness of the spacer structure also affects the lap shear strength. Since the spacer structure of VDH is bulkier than that of IDH, a lower melting transition was obtained for VDH, leading to the improved heat curing conversion of the epoxy resin. Moreover, the spacer structure of VDH is more flexible than that of IDH, owing to the fact that its load endurance is higher and its impact sensitivity is lower than that of IDH. Owing to these properties, the cured epoxy resin system using VDH could withstand higher loads with higher elongations. Therefore, the lap shear test results suggest that a hardener with a flexible and bulky spacer structure results in a cured epoxy resin system that exhibits excellent mechanical properties [29].

### 3.3. Contact Contamination of Liquid Crystals

**Figure 7:**
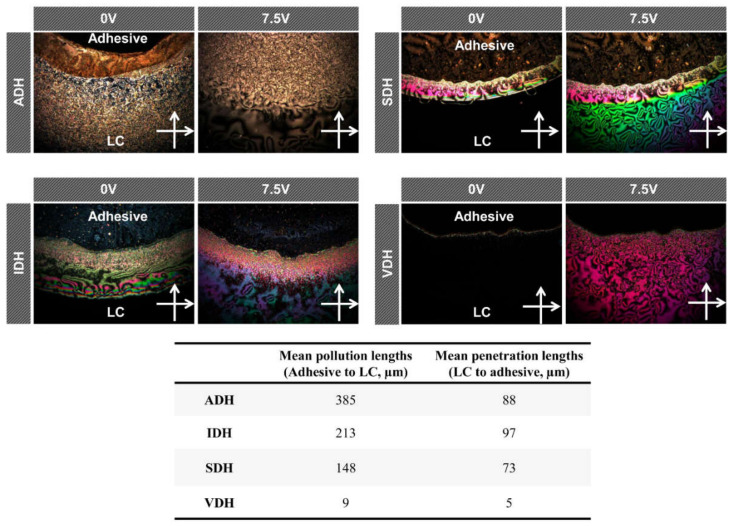
Contact contamination results of various epoxy resin systems. The liquid crystal (LC) texture area indicates contact contamination at 0 V.

During the LCD fabrication process, the sealant and LCs come in direct contact during the LC injection process. During the curing process, any unreacted components of the sealant may leak into the LCs, causing alignment defects in the final device [30,31]. For this reason, we evaluated whether the prepared epoxy-based sealing materials contaminated LCs on contact; the results are shown in Figure 7. Notably, the contamination results kept within the abovementioned conversion ratios. The epoxy resin system with VDH exhibited a high conversion ratio of 90.4%, which meant that a vast majority of dihydrazides and epoxy resins reacted during the curing process. Thus, the number of unreacted components of this system after the curing process would be low, resulting in an extremely low LC contamination level of approximately 9 µm. In contrast, the epoxy resin system formed using IDH showed the lowest conversion ratio of 73.9%, meaning that it contained a greater number of the unreacted components. As a result, some of these unreacted components leaked to the LC area, contaminating the LCs and causing defects in them (about 213 µm). The spacer structure of the dihydrazides also affected the extent of LC contamination. Although the melting points of SDH and ADH are similar, the contamination area of LCs was greater in the case of the ADH system (about 385 µm) because the alkyl chain of SDH is much longer than that of ADH. Thus, it can be concluded that, when the dihydrazide compound such as VDH has a low melting point and a bulky spacer structure, the heat conversion ratio is high, and the extent of LC contamination will be low under industrial heat-curing conditions. Furthermore, the cross-linking density of various epoxy resin systems can be compared by measuring the penetration lengths of the LCs into the adhesive material. As shown in Figure 7, the mean penetration lengths of the epoxy resin systems were in accordance with the heat curing conversions, and the epoxy resin system with VDH showed the lowest penetration length of approximately 5 µm, indicating the highest cross-linking density among the prepared sealing materials.

### 3.4. Electro-Optical Characteristics

**Figure 8:**
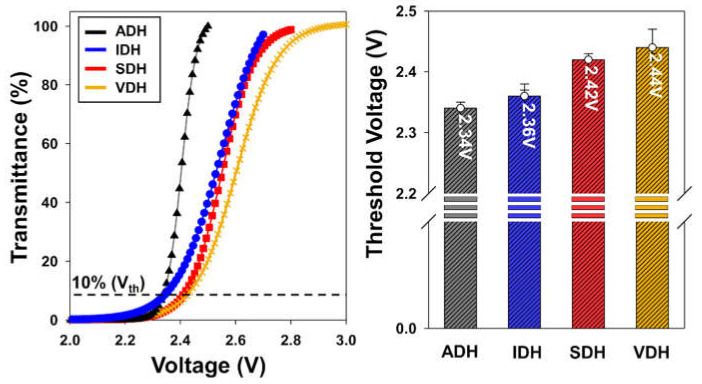
Voltage–transmittance curves for various epoxy resin systems and their threshold voltages.

With respect to the realization of high-quality LCDs, the electro-optical characteristics are of great importance. When a voltage is applied to the top and bottom electrodes, the vertically aligned LCs become horizontally aligned, and light can pass through the LCD cells. Thus, to evaluate the electro-optical characteristics of the epoxy resin systems, we determined their voltage–transmittance (V-T) curves and their threshold voltage (V_th_) values; the results are shown in Figure 8. In comparing the V-T curves of the epoxy sealing materials, it can be seen that the V_th_ values of the ADH and IDH systems are lower than those of the SDH and VDH systems. As shown in Figure 7, in the case of the epoxy system formed using ADH or IDH, the LC defect area stemming from cross contamination is large; this had an adverse effect on the vertical alignment of the LCs. Above the LC defect area, the LCs did not align vertically with ease, and they readily became horizontally aligned when a voltage was applied, leading to a decrease in the V_th_ value. In contrast, the epoxy resin systems formed using VDH or SDH exhibited stable V-T curves with relatively high V_th_ values because their contamination areas were smaller.

**Figure 9:**
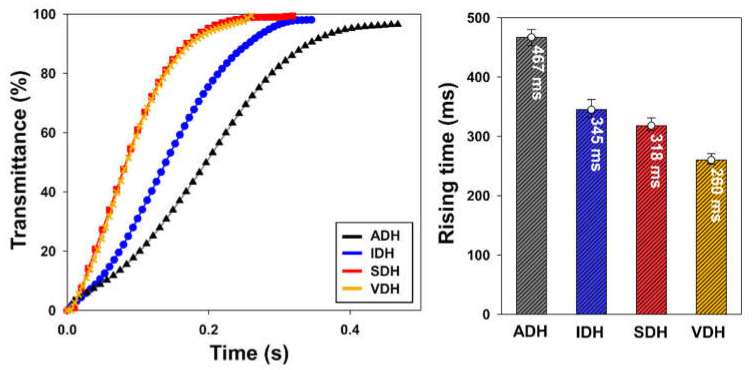
Response time curves for various epoxy resin systems and their rising response times.

Figure 9 shows the rising response time of each epoxy resin system. The epoxy sealing material formed using VDH exhibited the fastest rising time of 260 ms. In contrast, the ADH system showed the slowest rising time of 467 ms. The reason for these results is the same as that for the observed V_th_ values. The size of the contamination area affects the vertical alignment of the LCs, with a large contamination area resulting in the disturbed movement of the LCs when a voltage is applied. Thus, as the extent of LC contamination decreases, the electro-optical switching characteristics of the LC device show improvement. Consequently, it is confirmed that the epoxy-based sealing material formed using a VDH compound as a latent curing agent affords low LC contamination, a stable V-T curve, and a fast response time due to high heat conversion ratio and a bulky spacer structure [32].

## 4. Conclusions

In this study, we prepared epoxy-based sealing materials for LCDs using different types of dihydrazides as the latent curing agent to achieve high mechanical properties and excellent electro-optical performances. The characteristics of the latent curing agents were analyzed through DSC measurements. The results confirmed that the used dihydrazide compounds were suitable for use as latent curing agents for LCD sealing materials. We also confirmed the correlation between the melting point and the thermal curing conversion of the dihydrazide agents. Under industrial heat-curing conditions, VDH exhibited the lowest melting point and the highest heat conversion ratio, as determined by a FTIR analysis. Moreover, it was observed that, the higher the conversion ratio is, the lower the extent of LC contamination will be. This is because the number of unreacted components decrease with an increase in the conversion ratio. This also influences the alignment properties of the LCs. Further, based on the results of the adhesive and lap shear strength measurements, it was concluded that the spacer structure of the dihydrazides determined the mechanical properties of the cured epoxy resin systems. We found that as the bulkiness of the spacer structure increases, the impact sensitivity of the cured epoxy resin decreases and its flexibility increases. This results in desirable elongation properties and high adhesive strength when an external force is applied. Finally, we evaluated the electro-optical characteristics of each epoxy resin system formed under industrial heat-curing conditions. It was observed that as the LC contamination area decreases, the electro-optical properties, including the threshold voltage and response time, improve. This is because the LC contamination area affects the alignment properties of the LCs, causing them to become horizontally aligned. Thus, it can be concluded that dihydrazides, and especially VDH, can be used as effective latent curing agents for LCD sealants, owing to their high conversion ratios, low ability to contaminate LCs, high mechanical properties, and good electro-optical characteristics.

## Data Availability

Data is contained within the article.
